# Nondestructive Determination of Nitrogen, Phosphorus and Potassium Contents in Greenhouse Tomato Plants Based on Multispectral Three-Dimensional Imaging

**DOI:** 10.3390/s19235295

**Published:** 2019-12-01

**Authors:** Guoxiang Sun, Yongqian Ding, Xiaochan Wang, Wei Lu, Ye Sun, Hongfeng Yu

**Affiliations:** 1 College of Engineering, Nanjing Agricultural University, Nanjing 210031, China; yongqiand@njau.edu.cn (Y.D.); wangxiaochan@njau.edu.cn (X.W.); seurobot@njau.edu.cn (W.L.); sunye@njau.edu.cn (Y.S.); hongfengyu@njau.edu.cn (H.Y.); 2 Jiangsu Province Engineering Lab for Modern Facility Agriculture Technology & Equipment, Nanjing 210031, China

**Keywords:** NPK, greenhouse tomato, three-dimensional reconstruction, multispectral, high-throughput plant phenotyping

## Abstract

Measurement of plant nitrogen (N), phosphorus (P), and potassium (K) levels are important for determining precise fertilization management approaches for crops cultivated in greenhouses. To accurately, rapidly, stably, and nondestructively measure the NPK levels in tomato plants, a nondestructive determination method based on multispectral three-dimensional (3D) imaging was proposed. Multiview RGB-D images and multispectral images were synchronously collected, and the plant multispectral reflectance was registered to the depth coordinates according to Fourier transform principles. Based on the Kinect sensor pose estimation and self-calibration, the unified transformation of the multiview point cloud coordinate system was realized. Finally, the iterative closest point (ICP) algorithm was used for the precise registration of multiview point clouds and the reconstruction of plant multispectral 3D point cloud models. Using the normalized grayscale similarity coefficient, the degree of spectral overlap, and the Hausdorff distance set, the accuracy of the reconstructed multispectral 3D point clouds was quantitatively evaluated, the average value was 0.9116, 0.9343 and 0.41 cm, respectively. The results indicated that the multispectral reflectance could be registered to the Kinect depth coordinates accurately based on the Fourier transform principles, the reconstruction accuracy of the multispectral 3D point cloud model met the model reconstruction needs of tomato plants. Using back-propagation artificial neural network (BPANN), support vector machine regression (SVMR), and gaussian process regression (GPR) methods, determination models for the NPK contents in tomato plants based on the reflectance characteristics of plant multispectral 3D point cloud models were separately constructed. The relative error (RE) of the N content by BPANN, SVMR and GPR prediction models were 2.27%, 7.46% and 4.03%, respectively. The RE of the P content by BPANN, SVMR and GPR prediction models were 3.32%, 8.92% and 8.41%, respectively. The RE of the K content by BPANN, SVMR and GPR prediction models were 3.27%, 5.73% and 3.32%, respectively. These models provided highly efficient and accurate measurements of the NPK contents in tomato plants. The NPK contents determination performance of these models were more stable than those of single-view models.

## 1. Introduction

Crops cultivated in greenhouses have short life cycles, high production levels, and high fertilizer demands, and their soil nutrients experience high consumption rates and thus require high rates of supplementation [1,2,3]. During production, crops cultivated in greenhouses are prone to deficiencies in nutrient elements, such as nitrogen, phosphorus, and potassium (NPK), which severely affect the crop yield and economic benefits [2]. Highly efficient, precise, and nondestructive measurements of plant NPK contents are of great significance for rapidly and accurately monitoring and assessing crop growth conditions, precisely managing cultivation, and evaluating plant production [3].

The traditional conventional analysis of the plant NPK content commonly includes stoichiometry. The plant N content is usually determined by the Kjeldahl method. The P content is usually determined by phosphorus-vanadium-molybdenum yellow spectrophotometry and molybdenum-antimony anti-absorption spectrophotometry. The K content is usually determined by flame atomic absorption spectrophotometry. Stoichiometry has a high measurement accuracy but is destructive, costly, relatively time consuming, and requires complex operations. This method can no longer meet the demands for the accurate nondestructive measurement and management of crops cultivated in greenhouses in a high-throughput and periodic manner [2,3].

With the development of computer vision technologies, spectral imaging technologies and sensor technologies, the nondestructive determination of plant nutrients can be achieved optically. The parameters of a plant’s optical characteristics or electrical signal characteristics can be captured by optical sensors, and the plant nutrient contents can be indirectly predicted through modeling [4,5,6]. This mainly includes the following methods:

(i) Monocular vision imaging: the plants images are captured by a color camera [7,8,9] or scanner [10,11], the determination of plant nutrients based on monocular vision imaging mainly uses the characteristic parameters or combinations of two-dimensional (2D) images (such as [2*G* − (*R* + *B*)]/(*R* + *G* + *B*), *R* + *G* − *B*, etc) in RGB, HSV, YUV, and Lab color space models to establish models for determining the plant nutrients. This method very simple, but it has high requirements for the illumination environment during image collection, and the model has relatively low applicability. Plant nutrient measurements based on a scanner require that plant samples are collected for scanning, which is relatively inefficient, and the types of nutrients that can be measured are very limited [10,11].
(ii) Multispectral imaging: several characteristic bands are selected according to the sensitive characteristics of plant nutrients, such as one or more characteristic special bands in visible and near-infrared bands respectively [12,13,14]. According to the images or reflectance values of multiple characteristic bands of plants, the plant canopy nutrient prediction models are established. Most of the imaging areas are plant canopy images or band reflectance values. Generally, only one plant nutrient content can be measured at one time. The imaging area of this method is very limited, and the stability of the measurement models are greatly affected by the imaging area and the natural light environment.
(iii) Hyperspectral imaging: hyperspectral imaging is mainly used for the selection of the characteristic wavelengths of the plant nutrients [15,16,17]. The characteristic wavelengths that reflect the physicochemical properties of the materials are extracted. Multispectral imaging can be applied for the practical determination of plant nutrients [2]. Characteristic wavelengths are mainly extracted using the artificial neural network (ANN) [2], random frog (RF) algorithm [18], correlation coefficient (r) [18,19,20,21,22,23,24], principal component analysis (PCA) [25,26,27], successive projections algorithm (SPA) [26,27,28], uninformative variable elimination (UVE) [28], segmented principal components analysis (SPCA) [29], and competitive adaptive reweighted sampling (CARS) [28,30], etc. For the plant nutrient models established according to the characteristic band spectral reflectance or vegetation indices, the modeling methods are mainly divided into linear and nonlinear types. The linear correction methods include linear regression (LR) [31], multiple linear regression (MLR) [20,24], stepwise regression (SWR) [23], and partial least squares (PLS) [18,20,21,28,30,32], etc. The nonlinear correction methods include ANN [19,20,27,28,32], the support vector machine (SVM) [19,21], and RF [32]. Among them, PLS and ANN are the most widely applied algorithms.
(iv) Fluorescence imaging: fluorescence imaging system, according to the fluorescence induction curve of plant chlorophyll, collects the fluorescence images of plant leaves or canopy by controlling the intensity of laser source (such as measuring light, actinic light, saturation light), and extracts the fluorescence parameters of plant leaves, such as minimum fluorescence under dark adaptation *F_0_*, maximum fluorescence under dark adaptation *F_m_*, etc. Based on fluorescence imaging, models for determining a plant’s physiological information, such as nutrients, diseases, and stress, are established mainly from the plant’s fluorescence properties [33,34]. This technology has high requirements for the imaging environment, excitation light source, and imaging equipment. In addition, it has high operational requirements, uses very expensive equipment, and cannot be widely applied at a large scale.
(v) Proximal optical sensors: mainly includes chlorophyll meter, reflectance sensor and fluorescence-based flavonols meters. The representative chlorophyll meters mainly include SPAD-502 (Konica Minolta, Tokyo, Japan), DUALEX (Force-A, Orsay, France), MC-100 Chlorophyll Concentration Meter (Apogee Instruments Inc., Logan, UT, USA), etc. The representative reflectance sensor mainly include Crop Circle ACS 430 (Holland Scientific, Lincoln, NE, USA), MSR5/87/16R (CropScan Inc., Rochester, MN, USA), and GreenSeeker (Trimble Inc., Sunnyvale, CA, USA), etc. The fluorescence-based flavonols meters mainly include DUALEX and MULTIPLEX (Force-A), etc. These sensors are mainly used to measure the N content of plant leaves or canopy, which usually only measures the chlorophyll or NDVI value of a small region of the plant canopy at a time, and the plant nutrient distribution is uneven, particularly in the absence of nutrient elements [1,35,36].
(vi) Plant’s electrical signals: there are also methods for measuring plant K and P contents based on the plant’s electrical signals [37,38]. Plant electrical signals are weak and prone to interference from the measuring environment. This measurement method requires the insertion of probes into plant leaves or stems and is thus destructive. Therefore, it is difficult to use this method to measure plant nutrients at a large scale and periodically during actual production.

At present, plant nutrient measurements mainly extract characteristic parameters from 2D images of plants (most of which are images of plant leaves, seedlings or canopies) to establish nutrient determination models, regardless of whether monocular vision, multispectral, hyperspectral, or chlorophyll fluorescence imaging technologies are used to collect data. Proximal optical sensors are used, mainly extract the single point or small area plant nutrient. Due to the complex three-dimensional (3D) morphology of plants, especially for the large plants, it is difficult to ensure measurement accuracy and stability when the characteristics from single-view 2D plant canopy imagery or single point are used to establish models for determining plant nutrients. Methods for the 3D reconstruction of plants are relatively mature and are mainly based on 2D laser lidar [39], 3D laser lidar [40], Kinect sensor [41], multiview stereo reconstruction [42], and multicamera synchronous reconstruction [43] technologies. However, the reconstructed plant 3D point cloud model contains only coordinate information and RGB color information. Recently, researchers have begun to study the reconstruction of plants with multispectral 3D point cloud models. Itakura et al. [44] took plant images from 50 angles of view (AOVs), and based on the principle of structure from motion, they reconstructed a plant 3D point cloud model and used the normalized red value r/(r + g + b) to construct a chlorophyll estimation model. Zhang et al. [45] took multispectral images of the plant canopy from 24 AOVs to construct a plant multispectral 3D point cloud model and constructed regression models using GNDVI, NDVI, RVI, DVI, and SPAD data. However, the efficiency of multispectral 3D point cloud model reconstructing is relatively low, as it requires the collection of plant images from dozens of AOVs. Sun et al. [46] took plant RGB-D and multispectral images from four AOVs to reconstruct a plant multispectral 3D point cloud model. They also established a plant chlorophyll estimation model based on the NDVI, GNDVI, CIG, RCIG, and RVI etc, which provided a good technique for the high-throughput measurements of plant phenotypes. However, the reconstruction accuracy of the multispectral 3D point cloud model has not been quantitatively evaluated.

In this study, the 3D reconstruction technology and hyperspectral imaging technology will deeply integrated to realize the reconstruction of multispectral 3D point cloud model of plants, the NPK levels in tomato plants measurement models based on 3D multispectral features information were proposed. The characteristic wavelengths of NPK were selected based on the principal component weights, the correlation coefficients of the plant spectral reflectance, and the selection probability by RF. Multiview images of tomato plants were collected by synchronously using a SOC710 hyperspectral imager and a Kinect sensor. Based on the principles of Fourier transform, the spectral reflectance of the characteristic band taken by SOC710 hyperspectral imager was registered into the depth coordinates of a Kinect sensor. A Kinect sensor pose estimation and self-calibration method were established, enabling the unified transformation of the coordinate system of the multiview point cloud and the rough registration of multiview point clouds. Finally, the iterative closest point (ICP) algorithm was used for the precise registration of multiview point clouds [41,47], thus reconstructing a multispectral 3D point cloud model of tomato plants. The reflectance information of the multispectral 3D point cloud model of the tomato plant canopy was the input value, and chemical measures of the plant NPK contents were the output values. These values were used to establish a back-propagation artificial neural network (BPANN) of the NPK contents in tomato plants and prediction models by SVM regression (SVMR) and Gaussian process regression (GPR), which enabled highly efficient and accurate measurements of the NPK contents in the tomato plant. This measurement method can be extended to measure other physiological information of the plant, providing good technical support for high-throughput measurements of plant phenotypes. The method is of great significance for the development of plant phenomics and other research fields.

## 2. Materials and Methods

### 2.1. Sample Cultivation

On 5 March 2019, tomato seedlings (Fenguan No. l) were transplanted into the Venlo greenhouse at Nanjing Agricultural University. Sixty plant seedlings were cultivated. Galuku coir substrate and Yamazaki tomato formula nutrient solution, with pH values of 6.0–6.5 and an electrical conductivity (Ec) of 1.2 mS·cm^−1^, were used for cultivation. To obtain tomato plant samples at different nutrient levels, five different doses of the nutrient solution, corresponding to 25%, 75%, 100%, 150%, and 200% of the standard formula, were used [3,46], with 100% being the dose required for normal tomato growth. Twelve tomato plants were assigned to each dose. From the fifth to the eighth week after transplantation, tomato leaves were collected, and dried, and their NPK contents were determined. In addition, multiview hyperspectral and RGB-D images of each tomato plant were collected.

### 2.2. Instrument and Spectral Data Collection

**Figure 1:**
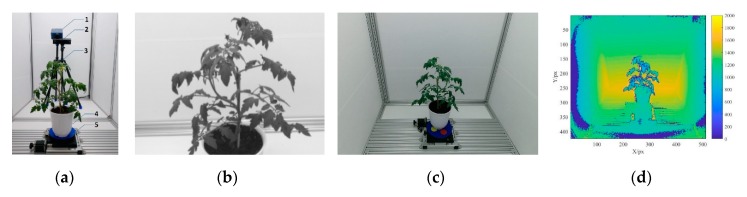
Multispectral three-dimensional reconstruction system for the greenhouse tomato plants. (**a**) Multispectral three-dimensional reconstruction system (1. SOC710 hyperspectral imager; 2. Kinect senor; 3. tripod; 4. tomato plant; 5. electric turntable); (**b**) spectral image taken at 451.6 nm by the SOC710 senor; (**c**) RGB image taken by the Kinect sensor; (**d**) depth image taken by the Kinect sensor.

This study established a multispectral 3D imaging system for greenhouse tomato plants, which mainly consisted of an imaging room, a light source, an SOC710 hyperspectral imager, a Kinect sensor, an electric turntable, a controller, and a graphics workstation (as shown in Figure 1a). The imaging room was made from aluminum materials, and its inner dimensions were 180 (length) × 120 (width) × 160 cm (height). The power of the light source was 150 W. The response wavelengths ranged from 380 nm to 2200 nm, and the light source had a stepless adjustable intensity. The SOC710 hyperspectral imager (SOC710 series hyperspectral imaging systems by Surface Optics Corporation, (San Diego, CA, USA) had a built-in scanning system, with a spectral range from 400 nm to 1000 nm and 128 wavelengths. The image resolution was 696 px × 510 px and the wavelengths for imaging could be set. The Kinect sensor V2.0 (Microsoft Corporation, Redmond, WA, USA) consisted of a color camera and a depth sensor. The resolutions of the RGB and depth images were 1920 px × 1080 px and 512 px × 424 px, respectively. The frame rate was 30 fps, with a detection range from 0.50 m to 4.50 m. The electric turntable disc had a diameter of 20.00 cm, and the angle ranged from 0° to 360°. The turntable disc was driven by a 57BYG stepper motor with a resolution of 0.0005° and a positioning precision of 0.01°. The controller used was an HW-36MT-3PG programmable controller (PRECHIN Corporation, Shenzhen, China) with 20 input interfaces, 16 output interfaces, a built-in three-channel, 100 kHz, high-speed pulse output, and an RS232C communication interface. The graphics workstation (HP Zhan 99 by Hewlett-Packard Corporation, Beijing, China) had an Intel(R) Xeon(R) E-2176M CPU @2.70 GHz, 32 GB memory, and an NVIDIA Quadro P600 4G graphics card. The system control software environment was jointly programmed using Visual Studio 2015 (Microsoft Corporation) and MATLAB 2017a (MathWorks Corporation, Natick, MA, USA) [46].

Figure 1b shows a spectral image taken by the SOC710 hyperspectral imager (using 451.6 nm as an example). Figure 1c shows an RGB image taken by the Kinect sensor. Figure 1d shows a depth image taken by the Kinect sensor. 

Collection of spectral and RGB-D images: for each tomato plant, an RGB-D image was taken by the Kinect sensor from four AOVs, with an angle interval of 90°. The four AOVs were represented by AOV1, AOV2, AOV3, and AOV4. Hyperspectral images were taken by the SOC710 hyperspectral imager and the Kinect sensor from four AOVs.

Collection of plant reference point cloud models: after collecting the tomato plant images, an Artec Eva handheld 3D scanner (with a scanning resolution of 0.1 mm) was used to scan the 3D model of each tomato. The point cloud model was preprocessed by Artec Studio software (Artec3D Corporation, Santa Clara, CA, USA). As the coordinate system differed between the scanning point cloud model and the reconstructed point cloud model, the same coordinate system was required before the reconstruction accuracy of the point cloud model could be evaluated. In this study, the key characteristic points of plants were manually selected to enable the alignment of the coordinates of the point cloud models.

Sample preparation for determining the plant NPK contents: after the tomato plant data were collected, the leaves were immediately placed in the oven at 100 °C, dried at 80 °C to constant weight, and stored in a desiccator for determination of the chemical values.

### 2.3. Multispectral 3D Point Cloud Modeling

The reconstruction of the multispectral 3D point cloud model of greenhouse tomato plants mainly consisted of four procedures: (i) Kinect sensor pose estimation and self-calibration; (ii) spectral reflectance registration; (iii) rough registration of the multiview point clouds; and (iv) precise registration of the multiview point clouds. The details of the procedures are described as follows:

(i) Kinect Sensor Pose Estimation and Self-Calibration

**Figure 2:**
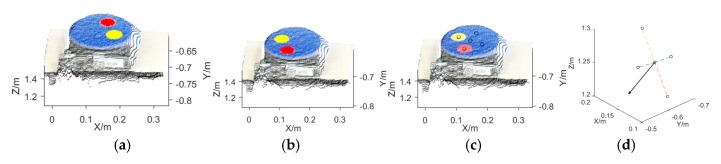
Pose estimation and self-calibration of the Kinect sensor. (**a**) Point cloud diagram of the turntable (rotation angle 0°); (**b**) point cloud diagram of the turntable (rotation angle 180°); (**c**) identified coordinates of the rotation axis; (**d**) normal vector of the rotation axis.

As shown in Figure 2a,b, before the plants’ multiview RGB-D and hyperspectral images were taken, two RGB-D images of the electric turntable (with a rotation angle of 180°) were obtained. The RGB-D image was converted into a 3D point cloud. According to the point cloud color threshold, the point cloud coordinates of the yellow and red calibration stickers were determined, and the center coordinates of the calibration sticker cloud coordinates were calculated. The center coordinates of the two RGB-D image calibration stickers were represented by Y1, R1, Y2, and R2. Figure 2c shows the central coordinates of the calibration stickers of the two RGB-D images and the central coordinates of the rotation axis *M* (*a_0_*, *b_0_*, *c_0_*). As shown in Figure 2d, the normal vector *P* of the rotary axis of the turntable was calculated and normalized to *P* (*a*, *b*, *c*). When the relative position of the Kinect sensor to the turntable was unchanged, pose estimation and self-calibration were not required, and the previously obtained calibration parameters *M* and *P* were used directly. Based on *M* and *P*, rough registrations of the multiview point clouds were performed.

(ii) Spectral Reflectance Registration

The reconstruction of the multispectral 3D point cloud model required the hyperspectral reflectance to be registered to the depth coordinate system of the Kinect sensor. As the SOC710 hyperspectral imager and the Kinect sensor took plant images from different positions and had different lens parameters, there were problems mainly with the displacement, rotation angle, and scaling coefficient transformation were associated with the two types of images.

In this study, the multispectral reflectance was registered to the depth coordinates according to the Fourier transform principles. Based on the Fourier spectra of the images, the translation, rotation matrix, and scaling coefficients of the images were calculated [46,48]. The translation between the Kinect sensor image *F* (*x, y*) and the SOC710 hyperspectral image *M* (*x, y*) was *M* (*x*-*x*_0_, *y*-*y*_0_), which could be calculated based on an inverse Fourier transformation of the cross-power spectrum. Fourier-Mellin transformation was used to transform the rotation (*θ*_0_) and scaling coefficient (*σ*) relationships in the Cartesian coordinate system into a translation relationship in the log-polar coordinates system [46].

**Figure 3:**
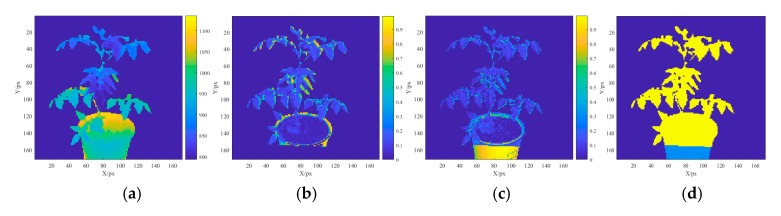
Spectral reflectance registration images. (**a**) Depth image for the ROI of the plant image; (**b**) ROI image after the spectral reflectance registration; (**c**) grayscale difference image for the ROI of the plant image; (**d**) spectrum coverage image.

Figure 3a shows a depth image taken by the Kinect sensor [the region of interest (ROI) of the plant image, blank background information]. Figure 3b shows a registered spectral reflectance profile (the ROI of the plant image). Figure 3c shows a normalized grayscale difference image for the ROI of the plant image based on the grayscale images taken by the Kinect sensor and the SOC710 imager (graying the RGB image taken by the Kinect sensor and the RGB image taken by the SOC710 imager). Figure 3d shows a binary image of the intersection of the multispectral grayscale image and the Kinect grayscale image for the ROI after the spectral reflectance registration. The area where the spectral reflectance image and Kinect grayscale image overlap was marked with yellow, and the nonoverlapping area was marked with pink. The quality of the spectral reflectance registration was evaluated based on the spectral coverage and grayscale similarity coefficient of the ROI of the plant image.

The relative positions between the SOC710 hyperspectral imager and the Kinect sensor were fixed; thus, it was not necessary to calibrate the transformation matrix repeatedly. In this study, the same transformation parameters were used for the spectral reflectance images in all wavelengths to maintain the consistency of spectral reflectance mapping. After the spectrum registration, the plant RGB image, depth image, and registered multispectral reflectance image were saved in a 3D array, which laid the foundation for reconstructing the plant multispectral 3D point cloud models.

(iii) Rough Registration of Multiview Point Clouds

First, according to the intrinsic parameters of the Kinect sensor, including the principal point coordinates (*c*_x_, *c*_y_) and focal length (*f*_x_, *f*_y_), the RGB-D images at various AOVs were converted into 3D point cloud images. Based on the ROI range, the ROI of the plant image was screened. Then, according to the center coordinates of the rotation axis *M* (*a*_0_, *b*_0_, *c*_0_), the point cloud at each AOV (*x_i_*, *y_i_*, *z_i_*) underwent a translation transformation, and the center of the rotation axis in the point cloud was translated to the origin of the Kinect coordinate system, as shown in Equation (1). According to the normal vector *P* (*a*, *b*, *c*) of the rotation axis, the translated point cloud (*x_i_^’^*, *y_i_^’^*, *z_i_^’^*) was rotated until its normal vector was rotated to the *Y*-axis. Finally, based on the actual rotation angle of each AOV, the inverse rotation angle γ around the *Y*-axis was determined, as shown in Equation (2). The coordinates of the point cloud (*X_i_*, *Y_i_*, *Z_i_*) in the unified coordinate system were obtained [46]:

(1) $$\left[\begin{array}{c} x_{i}^{\prime }\\ y_{i}^{\prime }\\ z_{i}^{\prime }\\ 1 \end{array}\right]=\left[\begin{array}{cccc} 1 & 0 & 0 & -a_{0}\\ 0 & 1 & 0 & -b_{0}\\ 0 & 0 & 1 & -c_{0}\\ 0 & 0 & 0 & 1 \end{array}\right]\left[\begin{array}{c} x_{i}\\ y_{i}\\ z_{i}\\ 1 \end{array}\right]=\left[\begin{array}{cccc} 1 & 0 & 0 & -a_{0}\\ 0 & 1 & 0 & -b_{0}\\ 0 & 0 & 1 & -c_{0}\\ 0 & 0 & 0 & 1 \end{array}\right]\left[\begin{array}{c} (i-c_{x})\times z_{i}/f_{x}\\ -(j-c_{y})\times z_{i}/f_{y}\\ Depth(i,j)/1000\\ 1 \end{array}\right]$$

(2) $$\left[\begin{array}{c} X_{i}\\ Y_{i}\\ Z_{i}\\ 1 \end{array}\right]=\left[\begin{array}{cccc} 1 & 0 & 0 & 0\\ 0 & \cos\alpha & -\sin\alpha & 0\\ 0 & \sin\alpha & \cos\alpha & 0\\ 0 & 0 & 0 & 1 \end{array}\right]\left[\begin{array}{cccc} \cos\beta & \sin\beta & 0 & 0\\ -\sin\beta & \cos\beta & 0 & 0\\ 0 & 0 & 1 & 0\\ 0 & 0 & 0 & 1 \end{array}\right]\left[\begin{array}{cccc} \cos\gamma & 0 & -\sin\gamma & 0\\ 0 & 1 & 0 & 0\\ \sin\gamma & 0 & \cos\gamma & 0\\ 0 & 0 & 0 & 1 \end{array}\right]\left[\begin{array}{c} x_{i}^{\prime }\\ y_{i}^{\prime }\\ z_{i}^{\prime }\\ 1 \end{array}\right]$$

where (*i*, *j*) are the coordinates of the depth image, (*c*_x_, *c*_y_) are the principal point coordinates of the Kinect sensor, (*f*_x_, *f*_y_) are the focal lengths of the Kinect sensor, *Depth* (*i*, *j*) is the depth value at coordinates (*i*, *j*) in mm, (*x_i_*, *y_i_*, *z_i_*) are the coordinates of the point cloud at the *i*-th AOV, (*x_i_^’^*, *y_i_^’^*, *z_i_^’^*) are the coordinates of the point cloud at the *i*-th AOV after translation transformation, (*X_i_*, *Y_i_*, *Z_i_*) are the coordinates of the point cloud at the *i*th AOV in the unified coordinate system, *α* is the angle between the normal vector projection on the YOZ plane and the *Y*-axis, in °, *β* is the angle between the normal vector projection on the XOY plane and the *Y*-axis in °, and *γ* is the rotation angle of the point cloud relative to the reference AOV in °. In this study, the first AOV for image collection was the reference AOV. (*a*_0_, *b*_0_, *c*_0_) were the center coordinates of the rotation axis.

(iv) Precise Registration of Multiview Point Clouds

Following the rough registration of the multiview point clouds, the point cloud models at various AOVs were transformed into a unified coordinate system. However, during the rotation of the plants, the plant stems and leaves experienced slight shaking. Therefore, the ICP algorithm was used to obtain the precise registration of the multiview point clouds. Details of the ICP registration procedures can be found in the literature [47,48]. ICP registration was performed for the point cloud model (*X_i_*, *Y_i_*, *Z_i_*) at each AOV successively; that is, the point cloud at the first AOV was ICP-registered with that at the second AOV, and the registration result was registered with the point cloud at the third AOV. This process continued until all the AOVs of the point cloud underwent precise registration, which yielded the plant 3D point cloud model.

As spectral reflectance registration was performed prior to the multiview point cloud registration, each spatial coordinate contained a multispectral reflectance value. Therefore, when the reconstruction of the plant 3D point cloud model was complete, the reconstruction of the multispectral 3D point cloud model was also achieved.

### 2.4. Determination of the Chemical Values of the NPK Nutrients

This study was based on the Agricultural Industry Standard of the People’s Republic of China (NY/T 2017-2011) for the determination of NPK in plants. The plant total N content was determined using the Kjeldahl method. The N content in the sample was expressed by the mass fraction *w_1_* in g/100 g. The total N content in the sample was calculated using Equation (3) as follows:

(3) $$w_{1}=\frac{(V_{2}-V_{0})\times c\times 0.0140}{m\times (V_{1}/V)}\times 100$$

where c is the concentration of the sulfuric acid standard titration solution (1/2 H_2_SO_4_) in 0.01 mol/L, *V*_2_ is the volume of the standard acid solution consumed by the sample in mL, *V*_0_ is the volume of the standard acid solution consumed by the blank in mL, *V*_1_ is the volume of liquid A to be tested during distillation in mL, *V* is the volume of liquid A to be tested in mL, *m* is the sample mass, in g, and 0.0140 is the mass of N in 1 mL of 1 mol/L sulfuric acid standard titration solution c (1/2 H_2_SO_4_) expressed in g.

The plant P content was determined by molybdenum-antimony anti-absorption spectrophotometry. The P content in the sample was expressed by the mass fraction *w_2_* in g/100 g. The total P content in the sample was calculated using Equation (4), as follows:

(4) $$w_{2}=\frac{\rho \times V}{m}\times \frac{V_{2}}{V_{1}}\times 10^{-4}$$

where *ρ* is the mass concentration of P in liquid A to be tested in mg/L, *V* is the volume of liquid A to be tested in mL, *V*_1_ is the dispensed volume of liquid A to be tested in mL, *V_2_* is the volume of the color solution in mL, and *m* is the sample mass in g.

The plant K content was determined by flame atomic absorption spectrophotometry. The K content in the sample was expressed by the mass fraction *w*_3_ in g/100 g. The total K content in the sample was calculated using Equation (5), as follows:

(5) $$w_{3}=\frac{(\rho -\rho_{0})\times V}{m}\times \frac{V_{2}}{V_{1}}\times 10^{-4}$$

where *ρ* is the mass concentration of K in liquid A to be tested in mg/L, *ρ_0_* is the mass concentration of K in the reagent blank digestion solution in mg/L, *V* is the volume of liquid A to be tested in mL, *V_1_* is the dispensed volume of liquid A to be tested in mL, *V_2_* is the volume of the color solution in mL, and *m* is the sample mass in g.

### 2.5. Data Processing and Analysis

#### 2.5.1. Optimal Wavelength Selection

**Figure 4:**
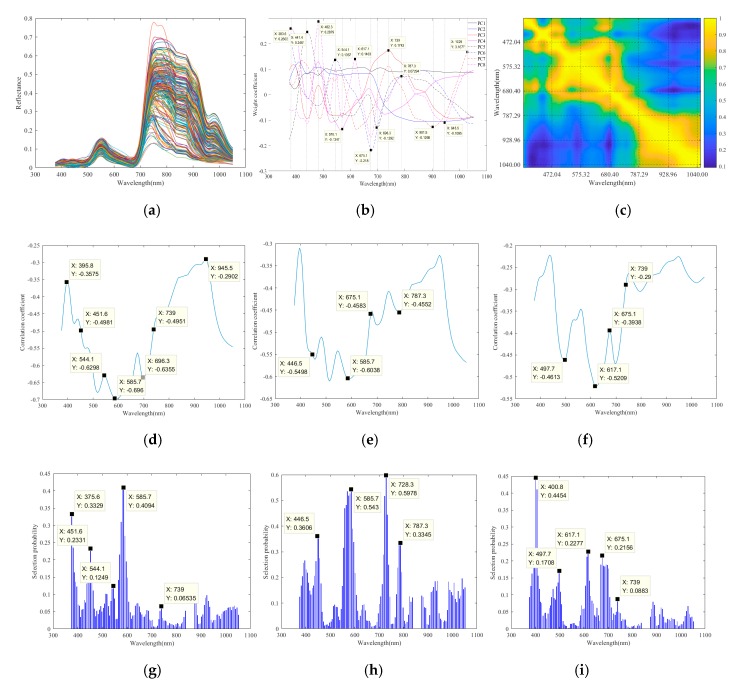
Characteristic wavelength selection of NPK. (**a**) Spectral reflectance curves; (**b**) principal component weights; (**c**) spectral reflectance autocorrelation coefficient; (**d**) correlation coefficient between the spectral reflectance and the N content; (**e**) correlation coefficient between the spectral reflectance and the P content; (**f**) correlation coefficient between the spectral reflectance and the K content; (**g**) RF selection probability of the N content characteristic wavelengths; (**h**) RF selection probability of the P content characteristic wavelengths; (**i**) RF selection probability of the K content characteristic wavelengths.

In this study, the spectral reflectance curves of the canopy ROIs of 60 tomato plants were selected. With four AOVs for each plant, there were a total of 240 leaf surface spectral reflectance curves. A Gaussian filter and data window No. 6 were used to smooth and denoise the spectral reflectance curve. Figure 4a shows the spectral reflectance curves of the tomato plants. The original spectral reflectance curves were in the range of 400 nm to 1100 nm, consisting of 128 wavelengths. In this study, due to the synchronous collection of data by the SOC710 hyperspectral imager and the Kinect sensor, the spectral reflectance data of six wavelengths, namely, 841.42, 846.85, 852.30, 857.74, 863.19, and 868.65 nm, were affected by the infrared interference of the Kinect sensor. Therefore, the statistics of these six wavelengths were removed, and only the spectral reflectance data of 122 wavelengths were used.

Wavelength selection method: (i) first, PCA was performed [25,26,27], during which the local peaks or valleys of the spectral reflectance principal component weights were observed to determine the positions of sensitive wavelengths. In this study, there were eight principal components, with the individual contributions of 50.02%, 30.72%, 8.13%, 4.69%, 2.77%, 1.64%, 0.82%, and 0.56% and a cumulative contribution of 99.35%. The principal component weights reached local peaks at 380.6, 441.4, 482.3, 544.1, 570.1, 617.1, 675.1, 696.3, 739.0, 787.3, 901.5, 945.5, and 1029.00 nm, as shown in Figure 4b. These results suggest that the spectral reflectance near these wavelengths was highly resolved. (ii) The correlation coefficients of the NPK contents with the spectral reflectance at each wavelength were separately calculated [18,19,20,21,22,23,24] to determine the wavelengths related to the NPK contents, as shown in Figure 4d–f. (iii) The characteristic wavelengths were selected based on the RF. Details of the algorithm steps can be found in the literature [18]. Figure 4g–i show the NPK wavelength selection probabilities. According to the spectral reflectance autocorrelation coefficient, as shown in Figure 4c, when the correlations of the spectral reflectances between adjacent wavelengths were highly significant, the wavelengths with local peaks in the selection were considered probable characteristic wavelengths in this study. The characteristic wavelengths of the N content were 375.6, 451.6, 544.1, 585.7, and 739.0 nm. The characteristic wavelengths of the P content were 446.5, 585.7, 728.3, and 787.3 nm. The characteristic wavelengths of the K content were 400.8, 497.7, 617.1, 675.1, and 739.0 nm. (iv) Based on the contributions of the principal components and correlation coefficients, 375.6 and 400.8 nm were removed.

PCA, CC, and RF were combined for the wavelength selection in this study. After using RF to select the characteristic wavelengths, the selection rationality was checked again. Based on PCA-CC, a characteristic wavelength of 696.3 nm was added to the N content, a characteristic wavelength of 675.1 nm was added to the P content, and a characteristic wavelength of 585.7 nm was added to the K content. Table 1 shows the selected characteristic wavelengths of the NPK contents.

#### 2.5.2. Evaluation of the Accuracy of Multispectral 3D Point Cloud Reconstruction

To verify the accuracy of the plant multispectral 3D point cloud reconstruction, the normalized grayscale similarity coefficient *D* and the degree of spectral overlap *C* of the registration area were used in this study to objectively evaluate the registration quality of the spectral reflectance. Equations (6) and (7) were used for the calculations. Based on the point cloud scans, this study quantitatively evaluated the accuracy of the multispectral 3D point cloud reconstruction, according to the Hausdorff distance set *HD* [49], as shown in Equation (8). The distribution ratio of *HD* and its average (*HD*_avg_), standard deviation (*HD*_std_) and maximum value (*HD*_max_) were computed to quantitatively evaluate the accuracy of the 3D point cloud reconstruction:

(6) $$D=1-\frac{\sum_{i=m}^{j=n}\left|M(i,j)-F(i,j)\right|}{m\times n\times 255}$$

(7) $$C=\frac{\sum_{i=m}^{j=n}F_{2}(i,j)\cap M_{2}(i,j)}{\sum_{i=m}^{j=n}F_{2}(i,j)}\times 100\%$$

(8) $$HD(RP,MP)=\underset{p_{a}\in RP}{D}\left\{\underset{p_{b}\in MP}{\min}\left\{d(p_{a},p_{b})\right\}\right\}$$

where *F* is the registered Kinect ROI image, *M* is the SOC710 image after registration, (*i*, *j*) are the image coordinates, *D* is the normalized grayscale similarity coefficient, *m* is the number of columns of registered region images, *n* is the number of rows of registered region images, *C* is the degree of spectral overlap of the ROI area in %, *F*_2_ is the Kinect ROI binary image, *M* is the SOC710 binary image after registration, *HD* is the minimum distance set of the reconstructed point cloud and the scanned point cloud, *RP* is the reconstructed set of points, *MP* is the scanned set of points, *p_a_* is a point within *RP*, and *p_b_* is a point within *MP*.

#### 2.5.3. NPK Model Construction and Evaluation

In this study, BPANN, SVMR, and GPR were used for the model construction. The independent variables of the constructed model were the spectral reflectance of the characteristic wavelengths of NPK. The average spectral reflectance values of the plant canopy 3D point cloud model and the average single-view 2D spectral reflectance values were selected. The dependent variables were the measured values of NPK. To evaluate the model accuracy, the coefficient of determination of the calibrated model R_c_^2^, the coefficient of determination of the predicted model R_p_^2^, the root mean square error of calibration (RMSEC), the root mean square error of prediction (RMSEP), and the relative error (RE) were used to assess the performance of the predictive models of NPK contents.

## 3. Results and Discussion

### 3.1. Multispectral 3D Point Cloud Modeling

**Figure 5:**
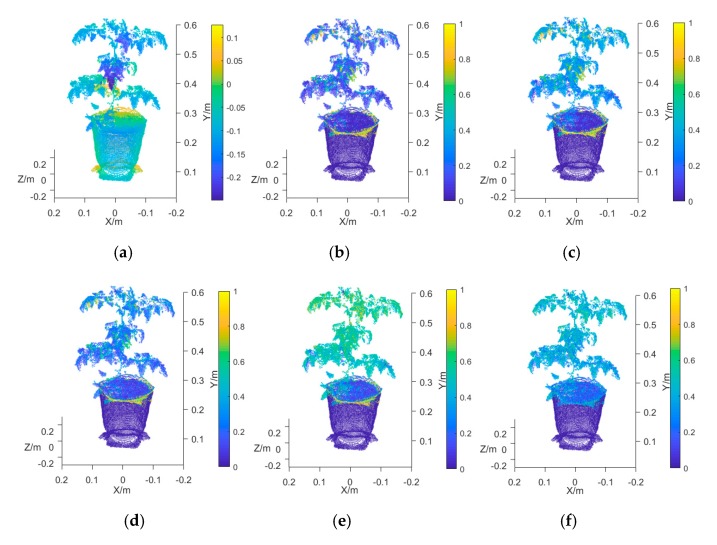
Multispectral 3D point cloud model of a tomato plant. (**a**) 3D point cloud model (depth); (**b**) point cloud model at 451.6 nm; (**c**) point cloud model at 544.1 nm; (**d**) point cloud model at 585.7 nm; (**e**) point cloud model at 696.3 nm; (**f**) point cloud model at 739.0 nm.

Based on the characteristic wavelengths of the plant NPK selected in Section 2.5.1 and the multispectral 3D point cloud model reconstruction procedures in Section 2.3, multispectral 3D point cloud models of tomato plants were reconstructed. Using the characteristic wavelengths of the N content as an example, Figure 5a–f show the 3D point cloud models of a tomato plant: the point cloud model at 451.6 nm, the point cloud model at 544.1 nm, the point cloud model at 585.7 nm, the point cloud model at 696.3 nm, and the point cloud model at 739.0 nm. Due to space limitations, the multispectral point cloud models of other plants and of P and K are not shown.

There were differences between the images of the SOC710 imager and the Kinect sensor. In this study, the Kinect sensor captured the plant canopy region (including the region of the cultivation pot underneath), but the SOC710 imager only collected the spectral reflectance of the plant canopy region. Therefore, the outer surface of the cultivation pot in the reconstructed 3D point cloud model had no spectral reflectance value. In this study, the ROI spectral reflectance value outside the SOC710 imaging region was blanked. As shown in Figure 5b–f, some regions of the cultivation pot body did not have reflectance values, but the point cloud coordinates in the plant canopy region all had spectral reflectance values.

### 3.2. Analysis of the Accuracy of the Multispectral 3D Point Cloud Reconstruction of Tomato Plants

#### 3.2.1. Evaluation of the Registration Quality of Spectral Reflectance

To verify the method of spectral reflectance registration proposed in this study, the normalized grayscale similarity coefficient *D* and the degree of spectral overlap *C* of the registration area, as shown in Equations (6) and (7), were used to objectively evaluate the registration quality of the spectral reflectance.

**Figure 6:**
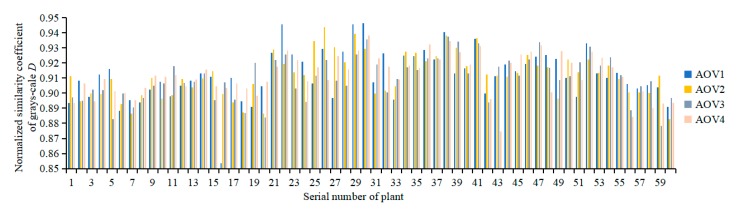
Normalized grayscale similarity coefficient.

Figure 6 shows the normalized grayscale similarity coefficient at various AOVs of 60 tomato plants (ROI of the plant image). Evaluation parameter *D* of the registration quality mainly evaluated the similarity between the registered SOC710 grayscale images and the Kinect grayscale images. The range of *D* was 0.8537–0.9465, and its average was 0.9116. This result indicated that the grayscale values of the registered images and the Kinect images were very similar, and the color correspondence after registration was good. As the multispectral reflectance images had the same transformation parameters, *D* could be used to evaluate the registration quality of reflectance. The grayscale differences produced in this study were mainly caused by the differences between the imaging regions captured by the SOC710 sensor and the Kinect sensor. As shown in Figure 3, the Kinect imaging region was larger than the SOC710 imaging region. In addition, there were apparent grayscale differences in the leaf margin regions of the plant canopy that were mainly due to the deformation produced by the SOC710 images after registration.

**Figure 7:**
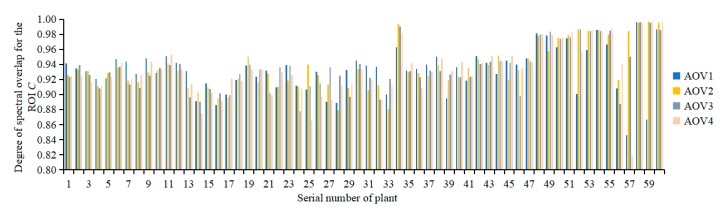
Degree of spectral overlap for the ROI.

Figure 7 shows the degree of spectral overlap of the ROI at various AOVs of 60 tomato plants. Evaluation parameter *C* of the registration quality mainly evaluated the degree of spectral overlap of the ROI of the plant image. The range of *C* was 0.8465–0.9969, and its average was 0.9343. The leaf surface of the plant canopy in the ROI was fully covered with multispectral reflectance information and was affected by the differences in the imaging regions of the two types of sensors. The lower parts of the cultivation pots had no spectral reflectance information. This problem can be solved by adjusting the sensor imaging regions based on the actual needs.

According to evaluation parameters *C* and *D* of the registration quality, the multispectral reflectance was registered to the Kinect depth coordinates based on the Fourier transform principles. As the Kinect sensor was influenced by the plant height and width, the measurement distance needed to be adjusted to ensure adequate measurement accuracy. Therefore, on the basis of determining the measurement position of the Kinect sensor, the SOC710 imager required the selection of appropriate lens parameters. This ensured that the requirements of the imaging angle and distance were met and that the measurement regions of the two imaging systems were appropriate to enhance the image registration quality.

#### 3.2.2. Evaluation of the Accuracy of Multispectral 3D Point Cloud Reconstruction

**Figure 8:**
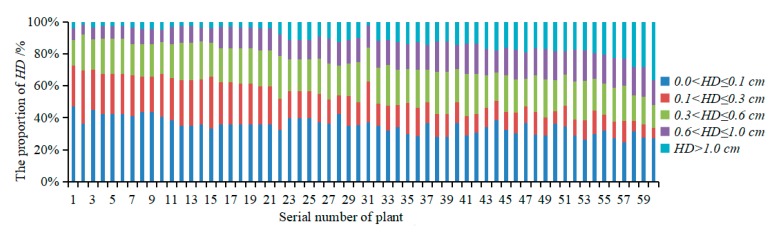
Proportions of *HD*.

To verify the accuracy of the plant multispectral 3D point cloud reconstruction proposed in this study, a Hausdorff distance set was used to objectively evaluate the accuracy of the 3D point cloud reconstruction. Figure 8 shows the proportions of *HD* in five segments: 0 cm < *HD* ≤ 0.1 cm, 0.1 cm < *HD* ≤ 0.3 cm, 0.3 cm < *HD* ≤ 0.6 cm, 0.6 cm < *HD* ≤ 1.0 cm, and *HD* > 1.0 cm. The average proportions of these segments were 34.98%, 17.98%, 21.52%, 16.17%, and 9.35%, respectively. These data indicated that, for the reconstructed point cloud and the scanned point cloud, the proportion of *HD* smaller than 0.6 cm was 74.48%, the proportion of *HD* smaller than 1.0 cm was 90.65%, and only 9.35% of *HD* was greater than 1.0 cm. This result indicated that most of the reconstructed 3D point cloud coordinates had errors smaller than 0.6 cm, and only a small number of point cloud coordinates deviated from their original coordinate positions.

**Figure 9:**
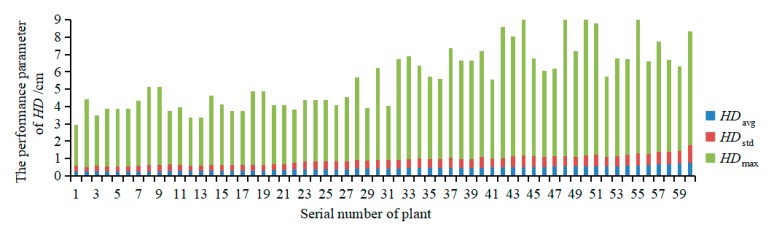
Performance parameters of *HD*.

Figure 9 shows the statistics of the performance parameters of distance set *HD*, including *HD*_avg_, *HD*_std_, and *HD*_max_. The range of *HD*_avg_ was 0.23–0.73 cm, and its average was 0.41 cm. The range of *HD*_std_ was 0.27–1.02 cm, and its average was 0.48 cm. The range of *HD*_max_ was 2.39–9.39 cm, and its average was 4.80 cm. *HD*_avg_ reflects the deviation distance of the entire reconstructed point cloud, *HD*_std_ reflects the dispersion of the deviation of the reconstructed point cloud, and *HD*_max_ reflects the maximum deviation distance of the reconstructed point cloud. The results indicated that the reconstructed point clouds of 60 plants had an average *HD*_avg_ of 0.41 cm and that the accuracy of the 3D point cloud reconstruction of the tomato plant was relatively high. Thus, the proposed 3D point cloud reconstruction method can be applied to tomato plants. There was still a small amount of noise, mainly because the plants were not rigid measurement objects, and slight shaking of plant stems and leaf surfaces would affect the accuracy of the RGB-D data during the multiview reconstruction. The requirements for data collection and the control of the imaging system are high.

The proposed method of plant multispectral 3D reconstruction can be applied to the multimodal 3D point cloud reconstruction of tomato plants but not to the 3D reconstruction of very small plants (such as seedlings) and plants with soft stems. As the Kinect sensor has limited accuracy, very small plant stems cannot be characterized. Soft plant stems shake during uniaxial rotation, preventing accurate 3D registration.

### 3.3. Construction of NPK Prediction Models

According to the characteristic wavelengths of NPK, multispectral 3D point cloud models of tomato plants were reconstructed. The average reflectance of the multispectral point cloud model (3DROI) of the plant canopy region was the input value, and the chemical measurements of the plant NPK were the output values. The NPK prediction models were separately constructed. Sixty tomato samples were used, of which 42 samples were used for modeling, and the remaining 18 samples were used to verify the model accuracy.

Based on the characteristic wavelengths of NPK, multispectral 3D point cloud models of tomato plants were reconstructed. For the multispectral point cloud model of the plant canopy region, three modeling approaches, namely, BPANN, SVMR, and GPR, were used to construct NPK prediction models of tomato plants. (i) In the BPANN model, the common three-layer structure was used. The activation function from the input layer to the hidden layer was a tansig function, and the activation function from the hidden layer to output layer was a purelin function. The learning function was a gradient descent function with momentum weight. The training function was the Levenberg-Marquardt function. The hidden layer had 15 nodes. The number of iterations was 500. The maximum number of confirmed failures was 15. The learning rate was 0.01, and the learning goal was 0.0001. (ii) For the SVMR model type, modeling was performed using the MATLAB R2017a Regression Learner APP, preset: fine Gaussian SVM; kernel function: Gaussian; kernel scale: 0.56; box constraint: automatic; epsilon: automatic; standardize data: true. (iii) For the GPR model type: modeling was performed using the MATLAB R2017a Regression Learner APP, preset: rational quadratic GPR; basis function: constant; kernel function: rational quadratic; use isotropic kernel: true; kernel scale: automatic; kernel sigma: automatic; sigma: automatic; standardize: true; optimize numeric parameters: true.

At the same time, the average value of the single-view multispectral reflectance of the point clouds of the plant canopy at AOV1, AOV2, AOV3, and AOV4 was the input value, and the chemical measurements of the plant NPK were the output values. The NPK prediction models based on single-view multispectral reflectance were constructed separately. With NPK as the three nutrients, BPANN, SVMR, and GPR as the three modeling approaches and AOV1, AOV2, AOV3, AOV4, and 3DROI as the five input methods, a total of 45 prediction models were constructed. The constructed models were saved, and the multispectral reflectance values corresponding to the 18 test samples were used as the input values to obtain the predicted plant NPK values.

### 3.4. Model Verification and Analysis

**Figure 10:**
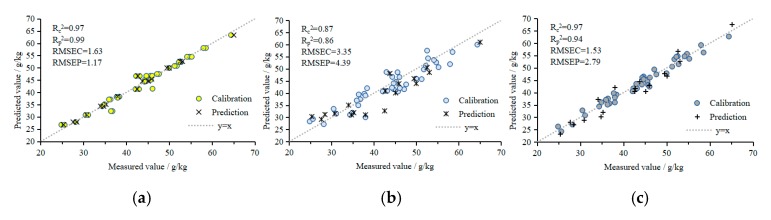

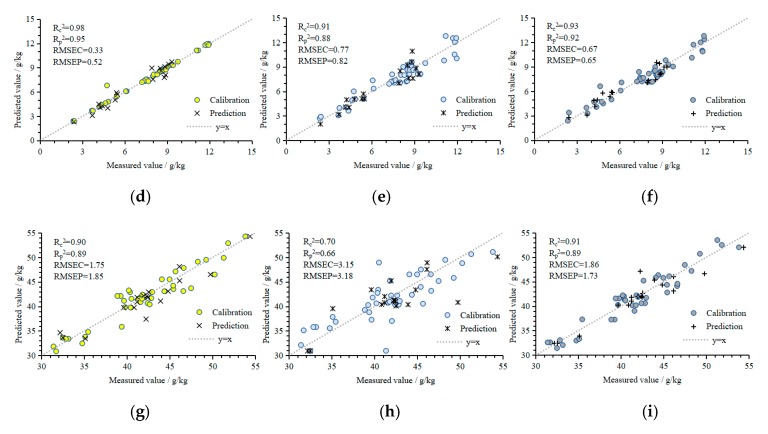
Correlations between the measured and predicted values of the NPK contents in tomato plants. (**a**) N content results predicted by the BPANN model; (**b**) N content results predicted by the SVMR model; (**c**) N content results predicted by the GPR model; (**d**) P content results predicted by the BPANN model; (**e**) P content results predicted by the SVMR model; (**f**) P content results predicted by the GPR model; (**g**) K content results predicted by the BPANN model; (**h**) K content results predicted by the SVMR model; (**i**) K content results predicted by the GPR model.

Figure 10 shows the results of the correlation analysis of the measured and predicted N, P, and K values of tomato plants based on the NPK prediction model using 3DROI multispectral reflectance values as the input values (due to space limitations, the figures showing the results of the correlation analysis of the measured and predicted values at other AOVs are not shown). Figure 10a–i shows the prediction results of the plant N, P, and K contents using the BPANN, SVMR, and GPR models, respectively.

Table 2 shows the evaluation results of the NPK prediction models:

(i) N models: 3DROI multispectral reflectance values were used as the input values for the N prediction models using BPANN, SVMR, and GPR. The R_c_^2^, R_p_^2^, RMSEC, and RMSEP of the BPANN prediction model were 0.97, 0.99, 1.63 mg/g, and 1.17 mg/g, respectively, and the RE was 2.27%. The R_c_^2^, R_p_^2^, RMSEC, and RMSEP of the SVMR prediction model were 0.87, 0.86, 3.35 mg/g, and 4.39 mg/g, respectively, and the RE was 7.46%. The R_c_^2^, R_p_^2^, RMSEC, and RMSEP of the GPR prediction model were 0.97, 0.94, 1.53 mg/g, and 2.79 mg/g, respectively, and the RE was 4.03%. The BPANN, SVMR, and GPR prediction models of the plant N contents all had good predictive performances. The performances of the BPANN and GPR models were similar, and were better than that of the SVMR model.
(ii) P models: 3DROI multispectral reflectance values were used as the input values for the P prediction models using BPANN, SVMR, and GPR. The R_c_^2^, R_p_^2^, RMSEC, and RMSEP of the BPANN prediction model were 0.98, 0.95, 0.33 mg/g, and 0.52 mg/g, respectively, and the RE was 3.32%. The R_c_^2^, R_p_^2^, RMSEC, and RMSEP of the SVMR prediction model were 0.91, 0.88, 0.77 mg/g, and 0.82 mg/g, respectively, and the RE was 8.92%. The R_c_^2^, R_p_^2^, RMSEC, and RMSEP of the GPR prediction model were 0.93, 0.92, 0.67 mg/g, and 0.62 mg/g, respectively, and the RE was 8.41%. The performance of the BPANN model was the best, followed by that of the GPR and SVMR models.
(iii) K models: 3DROI multispectral reflectance values were used as the input values for the K prediction models using BPANN, SVMR, and GPR. The R_c_^2^, R_p_^2^, RMSEC, and RMSEP of the BPANN prediction model were 0.90, 0.89, 1.75 mg/g, and 1.85 mg/g, respectively, and the RE was 3.27%. The R_c_^2^, R_p_^2^, RMSEC, and RMSEP of the SVMR prediction model were 0.70, 0.66, 3.15 mg/g, and 3.18 mg/g, respectively, and the RE was 5.73%. The R_c_^2^, R_p_^2^, RMSEC, and RMSEP of the GPR prediction model were 0.91, 0.89, 1.86 mg/g, and 1.73 mg/g, respectively, and the RE was 3.32%. The performance of the GPR model was the best, followed by that of the BPANN and SVMR models.

The statistical results indicated certain differences in the performances of the NPK prediction models using the multispectral reflectance values at AOV1, AOV2, AOV3, and AOV4 as the input values. Due to the complex 3D morphology of the plant, there were differences among the plant regions captured at different AOVs. Therefore, the model input values were different, and some models consequently had significantly lower performances than other models at different AOVs. For example, the performance of the SVMR prediction model for the N and K contents was significantly lower when the multispectral reflectance value at AOV3 was used as the input value than when that at any of the other AOVs was used as the input value.

When measuring the chemical values of plant nutrient contents, destructive sampling, drying, and measurements are necessary. Therefore, there are strict requirements for the collection area and weight of the samples. Chemical measurements often cannot specifically target a particular point of the plant or a particular leaf, especially for small plants. The entire plant canopy is often used as a sample for chemical measurements. Conventional plant nutrient prediction methods based on spectral imaging technology are often used to construct models using single-view plant spectral image information (mostly of the plant canopy). Due to the unevenness of the distribution of the plant nutrient contents, the actual measured values of the nutrients cannot be accurately determined, which leads to prediction models instability. The measurement region for plant nutrient contents needs to correspond one-to-one with the imaging region for the plant spectral information to construct prediction models with good performance. In this study, the entire plant canopy was the measurement region; therefore, it was reasonable to use the multispectral reflectance values of the plant canopy 3D point cloud model to construct the NPK prediction models. The statistical results indicated that, although the performance of several prediction models using single-view multispectral reflectance information at AOV1, AOV2, AOV3, and AOV4 as the input values was better than that of models using 3DROI values, the single-view point cloud information could not fully reflect the information of the entire plant canopy. Thus, the prediction models could not stably predict the plant nutrient contents. 3DROI included information on the entire plant canopy, so the NPK prediction models based on 3DROI had a better performance stability than the single-view models.

## 4. Conclusions

A registration method for images from heterogeneous sources based on the principles of Fourier transform was proposed. The multispectral reflectance was accurately registered to the depth coordinate system so that each depth coordinate had a multispectral reflectance value. The normalized grayscale similarity coefficient, and the degree of spectral overlap of the registration area were used to objectively evaluate the registration quality of the spectral reflectance. The results indicated that this method could accurately registered the multispectral reflectance images collected by the SOC710 imager to the RGB-D image coordinate system of the Kinect sensor. A 3D reconstruction method for multiview RGB-D images based on Kinect sensor pose estimation and self-calibration was proposed. Combined with the ICP precise registration algorithm, plant multispectral 3D point cloud models were reconstructed. The HD distance set was used to objectively evaluate the accuracy of the 3D point cloud reconstruction. The results indicated that the average distance set of the reconstructed point clouds of 60 tomato plants was 0.41 cm, suggesting that the reconstructed 3D point clouds had relatively high accuracy, which could be applied for the reconstruction of the 3D point clouds of tomato plants. The prediction models of the NPK contents of tomato plants using BPANN, SVMR, and GPR were constructed separately based on multispectral point cloud models. The results indicated that the accuracy of the prediction models using the 3D multispectral information of the plant canopy as the input value could predict NPK. The stability of the NPK prediction models using this approach was better than that of the single-view models. The accuracy of the plant 3D point cloud models was limited by the Kinect sensor. Through using multiple single-view RGB-D images, multiple 3D point cloud models could be merged to enhance the accuracy of the plant 3D point cloud models and to further improve the performance of the plant NPK prediction models. The plant multispectral 3D reconstruction and measurement systems proposed in this study are superior to the conventional measurements by 2D images and provide accurate and highly efficient methods for the high-throughput measurements of plant nutrient contents. This measurement method was not suitable for plants with soft stems or relatively small plants (such as seedling stage). Because plants were non-rigid measurement objects, the large shaking caused by rotation, as a result, multiview point clouds cannot be accurately registered. This method can be improved to multiview synchronous acquisition, but it will increase the cost of measurement equipment. These systems are easy to implement widely, have good application prospects, and are of great significance to the development of plant phenomics and other research fields.

## Figures and Tables

**Table 1:** Optimal wavelength selection of N, P, and K by the PCA-CC-RF method.

Nutrients	Method	Number	Characteristic Wavelength/nm
N	PCA-CC-RF	5	451.6, 544.1, 585.7, 696.3 *, 739.0
P	PCA-CC-RF	5	446.5, 585.7, 675.1 *, 728.3, 787.3
K	PCA-CC-RF	5	497.7, 585.7 *, 617.1, 675.1, 739.0

* denotes the values based on PCA-CC selection only; the values without an asterisk are based on PCA-CC-RF selection.

**Table 2:** Performance results based on different regression models.

Model		BPANN				SVMR				GPR			
	Input	Rc^2^	RMSEC	Rp^2^	RMSEP	Rc^2^	RMSEC	Rp^2^	RMSEP	Rc^2^	RMSEC	Rp^2^	RMSEP
N	AOV1	0.95	2.14	0.90	3.72	0.93	2.52	0.90	3.71	0.94	2.18	0.95	2.64
	AOV2	0.98	1.26	0.94	2.67	0.90	2.86	0.85	4.17	0.95	2.10	0.92	3.14
	AOV3	0.90	2.88	0.84	4.25	0.73	4.87	0.77	4.93	0.96	1.94	0.95	2.65
	AOV4	0.92	2.56	0.93	2.77	0.83	3.84	0.81	4.68	0.98	1.32	0.94	3.04
	3DROI	0.97	1.63	0.99	1.17	0.87	3.35	0.86	4.39	0.97	1.53	0.94	2.79
P	AOV1	0.94	0.69	0.92	0.85	0.90	0.86	0.87	0.82	0.93	0.67	0.93	0.62
	AOV2	0.98	0.37	0.95	0.55	0.91	0.81	0.90	0.72	0.94	0.65	0.92	0.65
	AOV3	0.98	0.41	0.93	0.61	0.89	0.84	0.84	0.95	0.93	0.68	0.90	0.71
	AOV4	0.97	0.57	0.92	1.00	0.89	0.90	0.90	0.98	0.94	0.97	0.90	1.10
	3DROI	0.98	0.33	0.95	0.52	0.91	0.77	0.88	0.82	0.93	0.67	0.92	0.65
K	AOV1	0.87	1.98	0.89	1.88	0.69	3.12	0.60	3.51	0.90	1.77	0.89	1.79
	AOV2	0.88	1.96	0.84	2.52	0.75	2.82	0.71	3.12	0.94	1.47	0.90	1.93
	AOV3	0.86	2.14	0.87	2.02	0.58	3.56	0.52	4.84	0.93	1.61	0.88	1.87
	AOV4	0.93	1.44	0.88	1.94	0.66	3.38	0.64	3.52	0.92	1.62	0.91	1.62
	3DROI	0.90	1.75	0.89	1.85	0.70	3.15	0.66	3.18	0.91	1.86	0.89	1.73
